# An *in vitro* model of human neocortical development using pluripotent stem cells: cocaine-induced cytoarchitectural alterations

**DOI:** 10.1242/dmm.017251

**Published:** 2014-10-02

**Authors:** Abigail A. Kindberg, Raphael M. Bendriem, Charles E. Spivak, Jia Chen, Annelie Handreck, Carl R. Lupica, Jinny Liu, William J. Freed, Chun-Ting Lee

**Affiliations:** 1 Cellular Neurobiology Research Branch, Intramural Research Program (IRP), National Institute on Drug Abuse, National Institutes of Health (NIH), Department of Health and Human Services (DHHS), Baltimore, MD 21244, USA; 2 Department of Pharmacology, Toxicology, and Pharmacy, University of Veterinary Medicine, Hannover 30173, Germany; 3 Center for Bio/Molecular Science & Engineering, US Naval Research Laboratory, Washington, DC 20375, USA

**Keywords:** Neocortical development, Dorsal forebrain model, hPSCs, Cocaine, Premature neuronal differentiation

## Abstract

Neocortical development involves ordered specification of forebrain cortical progenitors to various neuronal subtypes, ultimately forming the layered cortical structure. Modeling of this process using human pluripotent stem cells (hPSCs) would enable mechanistic studies of human neocortical development, while providing new avenues for exploration of developmental neocortical abnormalities. Here, we show that preserving hPSCs aggregates – allowing embryoid body formation – while adding basic fibroblast growth factor (bFGF) during neuroepithelial development generates neural rosettes showing dorsal forebrain identity, including Mash1^+^ dorsal telencephalic GABAergic progenitors. Structures that mirrored the organization of the cerebral cortex formed after rosettes were seeded and cultured for 3 weeks in the presence of FGF18, BDNF and NT3. Neurons migrated along radial glia scaffolding, with deep-layer CTIP2^+^ cortical neurons appearing after 1 week and upper-layer SATB2^+^ cortical neurons forming during the second and third weeks. At the end of differentiation, these structures contained both glutamatergic and GABAergic neurons, with glutamatergic neurons being most abundant. Thus, this differentiation protocol generated an hPSC-based model that exhibits temporal patterning and a neuronal subtype ratio similar to that of the developing human neocortex. This model was used to examine the effects of cocaine during neocorticogenesis. Cocaine caused premature neuronal differentiation and enhanced neurogenesis of various cortical neuronal subtypes. These cocaine-induced changes were inhibited by the cytochrome P450 inhibitor cimetidine. This *in vitro* model enables mechanistic studies of neocorticogenesis, and can be used to examine the mechanisms through which cocaine alters the development of the human neocortex.

## INTRODUCTION

The cerebral cortex represents the most complex, and uniquely human, organ structure. Cerebral cortical diseases are diverse, spanning from developmental disorders to neurodegenerative conditions in aging adults. In addition, the cerebral cortex is highly susceptible to both physical injury and toxin-induced disorders of brain development. The majority of our knowledge of the cerebral cortex has been gained through the use of animal models but, although the neocortex is similarly organized in all mammals, the complexity of human cerebral cortical development greatly exceeds that of any animal (Rakic, 2009; Hill and Walsh, 2005). Therefore, a human-cell-based *in vitro* neocortical model is potentially one of the most important model systems that could be developed from human pluripotent stem cells (hPSCs).

Adverse consequences of cocaine on human fetal brain development remain unclear owing to confounding factors such as time and dose of cocaine exposure, prenatal health, and exposure to other drugs. Although the overall effect of cocaine is modest (Singer et al., 2002; Singer et al., 2004), a number of studies have shown that exposure to higher doses of cocaine results in substantial neurological deficits (Chiriboga et al., 2007; Tronick et al., 1996). In primates, prenatal exposure to cocaine during the second trimester, when neural progenitor proliferation is most active, has been shown to cause cytoarchitectural changes in the neocortex (Lidow et al., 2001). Previously, we demonstrated that prenatal cocaine exposure, during active neural progenitor proliferation, altered the distribution of glutamate and GABAergic neurons in the developing rat neocortex (Lee et al., 2011). We also showed that cytochrome-P450-dependent reactive oxygen species (ROS) formation, which occurs during cocaine metabolism, is responsible for cocaine-induced cell-cycle arrest in rat neuroprogenitor cells (Lee et al., 2008). However, how cocaine-induced changes in neuroepithelial (NE) cell-cycle kinetics are related to cocaine-induced alterations in neocortical development is unknown. In addition, substantial species differences in cytochrome-P450-mediated drug metabolism have been reported (Martignoni et al., 2006); therefore, information derived from our previous studies using animal models can only be extrapolated to humans to a limited degree.

The mammalian cerebral cortex consists of two main types of neurons, excitatory glutamatergic projection neurons and inhibitory GABAergic neurons. Roughly 80% of cortical neurons are glutamatergic and are generated by dorsopallial cortical progenitor cells. Inhibitory GABAergic interneurons make up the remaining 20% of cortical neurons, and are produced in the ganglionic eminence of the ventral telencephalon, but migrate to the neocortex during development (Rakic, 2009). The glutamatergic neurons of the cortex are produced in an organized and predictable temporal order to make up the six layers present in the mature cerebral cortex, with deep-layer neurons being generated first and upper-layer neurons being generated last (Rakic, 2009). Importantly, in higher primates, the number and complexity of GABAergic neurons increases proportionally to glutamatergic neurons (Jones, 2009). A lineage of neocortical GABAergic neurons expressing Mash1, Dlx1 and Dlx2, which are generated from Mash1-expressing progenitors, in the dorsal forebrain ventricular and subventricular zone (VZ/SVZ) has been reported in humans (Letinic et al., 2002). However, others have shown that the majority of GABAergic interneurons in the human neocortex, similar to rodent GABAergic neuron formation, are derived from the ventral forebrain medial and caudal ganglionic eminences (Ma et al., 2013). Therefore, the origin of these GABAergic neocortical interneurons in humans and primates has not been firmly established.

**RESOURCE IMPACT**

**Background**

One of the major areas of biomedical science for which the knowledge gained from animal models is still limited is the study of the normal and diseased cerebral cortex. This is because, although similarities are shared among mammals, the complexity of the human cerebral cortex greatly exceeds that of any other animal. The human cerebral cortex is affected by a wide spectrum of disorders, including developmental and genetic defects, drug- and environmental-toxin-induced disorders, traumatic injury, and neurodegenerative diseases. Human pluripotent stem cells represent a unique system to model the development of the human cerebral cortex, but current models do not always replicate the major types of neurons and developmental processes that are present in the human cortex, which might also limit quantitative observations. A reproducible *in vitro* model of human neocortical development could facilitate the development of new drugs for reversing or preventing disorders of the cerebral cortex.

**Results**

This study describes a model of human neocortical development, based on the use of human pluripotent stem cell aggregates without cell dissociation, and inclusion of key trophic factors. This model preserves important features of human neocortical development, including the presence of radial glia scaffolding that supports neuronal migration, the sequential development of deep- and upper-layer neurons, and the formation of glutamatergic and GABAergic neurons, which are generated from the dorsal neuroepithelium in approximately the same proportion as found in the human neocortex. This model was used to examine the developmental effects of cocaine, the developmental toxicity of which has been particularly difficult to examine in humans because of the presence of other co-existing factors that can affect cortical physiology, including poor environmental and nutritional conditions. In this study, cocaine was found to induce oxidative stress, cause premature neuronal differentiation and impair neocortical patterning. Notably, these changes were reversed by the cytochrome P450 inhibitor cimetidine, which blocked cocaine-induced oxidative stress.

**Implications and future directions**

This model showed that the use of cimetidine, or similar drugs, might prevent cocaine-induced brain developmental deficits. However, further investigations are needed to test the feasibility of this approach, in particular gaining more *a priori* knowledge of the mechanisms of cocaine abuse. The identification of the mechanisms and the critical developmental periods for cocaine-induced brain injury could aid in preventing and alleviating its consequences. Moreover, because the present model is highly reproducible and amenable to drug screening, it could be employed to identify strategies for preventing or ameliorating other conditions of developmental injury to the human cerebral cortex. In the future, the specific isoforms of cytochrome P450 that are responsible for cocaine-induced impairment of neocortical development could be identified using the present, or similar, *in vitro* models.

The quantity and diversity of GABAergic interneurons largely determines the complex functions of the neocortex (Markram et al., 2004; Gupta et al., 2000). Various protocols exist for modeling cortical development from PSCs; however, these protocols do not replicate the production of inhibitory interneurons from dorsal cortical ventricular progenitors, and therefore might not entirely model the complexity of the human cerebral cortex (Shi et al., 2012; Gaspard et al., 2008). These studies have attained the temporal generation of deep-layer and upper-layer neurons; however, the organized human cortical structure, including scaffolding support of the migrating cortical neurons, has still not been completely accomplished.

Problems constructing human cortical structures could be due to the methods used in currently available protocols (Shi et al., 2012; Chambers et al., 2009), in which hPSCs are dissociated and then allowed to re-aggregate later. This dissociation disrupts intercellular communications between hPSCs, which might result in the loss of the ability of hPSCs to differentiate to multi-lineage neocortical cells. We hypothesize that preserving hPSC aggregates – allowing embryoid body (EB) formation – and supplementing the medium with defined neocortical trophic factors, will enable the differentiation of hPSCs to structures resembling the human neocortex *in vitro*. Defined neocortical trophic factors – including basic fibroblast growth factor (bFGF), which is known to play a role in establishing the dorsal neuroepithelium of the anterior neocortex (Raballo et al., 2000), maintaining the proliferative NE pool (Israsena et al., 2004) and priming NE cells with a radial glial identity (Yoon et al., 2004) – were used to supplement conversion from embryonic stem (ES) to NE cells. In addition, FGF18, BDNF and NT3, which have been shown to be involved in neocortical patterning and development (Hasegawa et al., 2004; Ohtsuka et al., 2013; Castellani and Bolz, 1999; Fukumitsu et al., 2006; Gorski et al., 2003), were used later in cortical neuronal differentiation. The resulting model, recapitulating the essential features of *in vivo* neocortical development, can be used to construct *in vitro* models of cocaine-induced neocortical disorders and develop therapeutic strategies for the prevention of cocaine-induced impairment of brain development. Additionally, a complex and temporally accurate dorsal forebrain cortical model generated from hPSCs could provide novel approaches for studying other cerebral cortex diseases.

## RESULTS

### Modeling of neocortical development from hPSCs

**Fig. 1.**
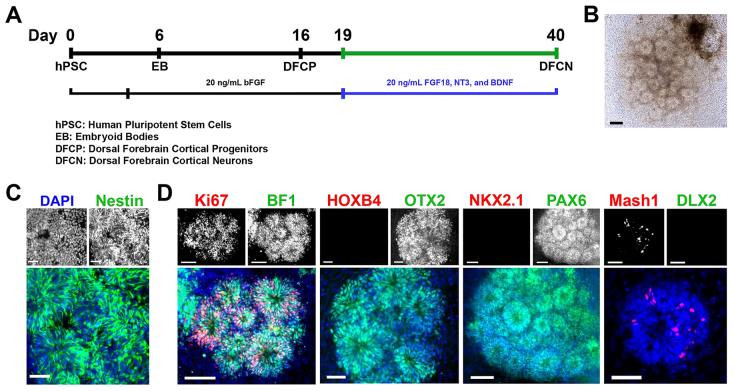
**Generation of neocortical NE rosettes from hPSCs.** (A) Schematic representation of the dorsal telencephalic cortical differentiation procedure. (B) Phase-contrast image of NE rosettes derived from hPSCs on day 16. Scale bar: 100 μm. (C) Nestin^+^ NE rosettes on day 16. Scale bars: 50 μm. (D) Dorsal forebrain NE cells generated from hPSCs. Expression of Ki67 and BF1 (scale bars: 100 μm), HOXB4 and OTX2 (scale bars: 50 μm), NKX2.1 and PAX6 (scale bars: 100 μm), and Mash1 and DLX2 (scale bars: 50 μm) by immunocytochemistry at day 16. Cell line: ES04.

We developed an hPSC-based neocortical differentiation protocol as shown in Fig. 1A. bFGF was included in cultures through the late EB to NE stage (day 5–16) to generate dorsopallial NE cells. Nestin^+^ NE rosettes with radially organized columnar cells were generated at the colony centers (Fig. 1B,C). NE cells in the rosettes were proliferative, as they expressed Ki67, a cell proliferation marker (Fig. 1D). Additionally, the NE rosettes expressed the anterior telencephalic transcription factors BF1 and OTX2 (Li et al., 2009), and the dorsal forebrain marker PAX6 (Manuel and Price, 2005) (Fig. 1D). Conversely the rosettes did not express the hindbrain and spinal cord marker HOXB4 and ventral telencephalic marker NKX2.1 (Fig. 1D). Also seen in the NE rosettes was a small population of Mash1^+^ DLX2^−^ NE cells (Fig. 1D), demonstrating the presence of dorsopallial GABAergic progenitor cells (Letinic et al., 2002).

**Fig. 2.**
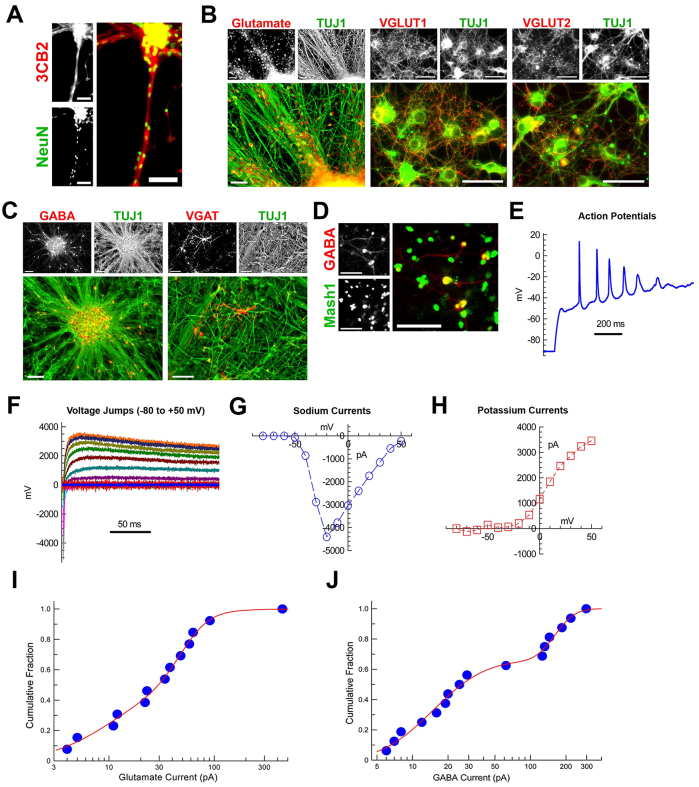
**Characterization of dorsal telencephalic cortical neurons.** (A) Expression of NeuN and 3CB2 by radial glial projections using immunocytochemistry at day 22. Scale bars: 50 μm. (B–J) Data from neocortical culture at day 40. (B) Expression of glutamate, VGLUT1, VGLUT2 and TUJ1 by immunocytochemistry. Scale bars: 50 μm. (C) Expression of GABA and TUJ1 (scale bars: 100 μm), and VGAT and TUJ1 (scale bars: 50 μm) by immunocytochemistry. (D) Expression of GABA and Mash (scale bars: 50 μm) by immunocytochemistry. (E–J) Electrophysiological analysis of dorsal forebrain cortical neurons generated from hPSCs. (E) Cortical neurons elicit action potentials when current clamped and injected with a current step. (F) Current traces of the cell when held at various membrane potentials under voltage clamp. Colors represent different clamp voltages. The peak amplitudes of the inward sodium currents and outward delayed rectifier potassium currents are plotted in panels G and H, respectively. (I,J) The responses to 100 μM glutamate (I) and to 10 μM GABA (J) were plotted as cumulative distributions, and both appeared as bimodal lognormal distributions. The responses to glutamate (I) showed one component (61%) with mean of 13 pA and a second component with mean of 50 pA. The responses to GABA (J) showed one component (66%) with a mean of 15 pA and a second component with a mean of 162 pA. Cell line: ES04.

On day 16, NE rosettes were manually picked up, maintained in suspended culture with bFGF for 3 days, and subsequently seeded on poly-ornithine/laminin-coated plates. Once seeded, the cultures were supplemented with neocortical trophic factors FGF18, BDNF and NT3 for 3 weeks in order to generate organized neocortical structures (Fig. 1A). Three days after NE rosettes were seeded, 3CB2^+^ radial glia projections extended from the colonies, providing architectural support for the migration of newly generated neurons from the NE (Fig. 2A), resembling the development of the neocortex *in vivo*. At the end of differentiation, on day 40, the majority of TUJ1^+^ neurons (82.8±7.8%; *n*=3) had adopted a glutamatergic phenotype, expressing glutamate (Fig. 2B), whereas a smaller percentage of TUJ1^+^ neurons (13.2±3.4%; *n*=3) were GABAergic, expressing GABA (Fig. 2C). Furthermore, expression of the vesicular glutamate transporters VGLUT1 and VGLUT2 (Fig. 2B) and the vesicular GABA transporter VGAT (Fig. 2C) were observed. In addition, GABAergic neurons coexpressed Mash1, suggesting that the neocortical GABAergic neurons were derived from the Mash1^+^ NE cells of the colonies (Fig. 2D).

We employed conventional whole-cell patch-clamp methods to evaluate the electrical excitability and the expression of receptors of the neurotransmitters glutamate and GABA. We found that, after 40 days of differentiation, cells that were held under current-clamp conditions exhibited robust action potentials (Fig. 2E) corresponding to large voltage-gated sodium currents and delayed rectifier potassium currents revealed under voltage-clamp conditions (Fig. 2F–H). Superfusion of glutamate or GABA also induced currents (Fig. 2I,J). These results demonstrate that cortical neurons derived from hPSCs develop both electrical excitability and reactivity to the two predominant neurotransmitters of the cerebral cortex.

### Temporal modeling of neocortical development

**Fig. 3.**
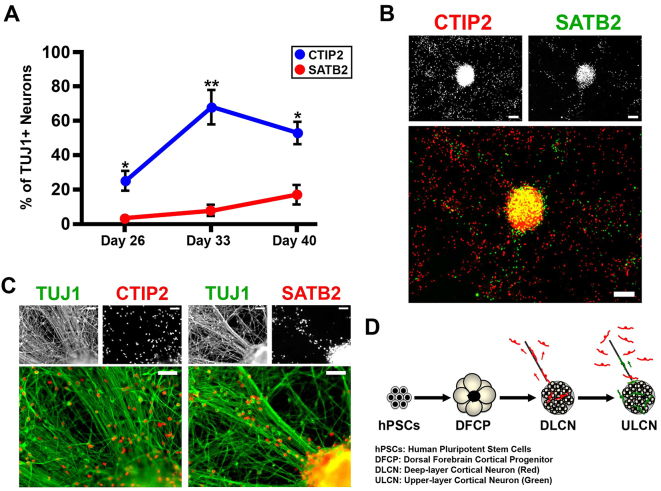
**Temporal ordering of dorsal forebrain cortical neurons generated from hPSCs.** (A) Time course tracing the generation of deep-layer CTIP2^+^ neurons and upper-layer SATB2^+^ neurons, during the final 3 weeks of differentiation. Statistical results comparing CTIP2 neuron generation and SATB2 neuron generation at each time point; *n*=3. *P<0.05, **P<0.01. Error bars, s.e.m. (B) Expression of the cortical layer markers CTIP2 and SATB2 by immunocytochemistry at day 40. Scale bars: 100 μm. (C) Characterization of dorsal cortical neurons by CTIP2 and TUJ1, and SATB2 and TUJ1, expression by immunocytochemistry at day 40. Scale bars: 50 μm. (D) Schematic illustrating hPSC differentiation to dorsal forebrain cortical progenitors and at the end of differentiation to deep (red)- and upper (green)-layer dorsal forebrain cortical neurons. Cell line: ES04.

Glutamatergic projection neurons in the mature cortex are generated in a fixed temporal manner. To examine sequential neocortical neurogenesis in our model, we studied the expression of CTIP2, a deep-layer cortical neuron marker, and SATB2, an upper-layer cortical neuron marker, over the final 3 weeks of differentiation. Deep-layer CTIP2^+^ neurons appeared on day 22, whereas upper-layer SATB2^+^ neurons differentiated later, beginning on day 33 (Fig. 3A and supplementary material Fig. S1). At the end of differentiation, on day 40, CTIP2^+^ neurons were evenly dispersed around the colonies, whereas SATB2^+^ neurons were aligned along the extended radial projections (Fig. 3B,C). These data indicate that CTIP2^+^ neurons were generated earlier, migrating outwards and dispersing from the colonies, whereas SATB2^+^ neurons exhibited later migration along the radial glial fibers (Fig. 3D).

### Modeling neocorticogenesis in 3D space

To generate a 3D hPSC-based neocortical model, rosettes were embedded in collagen gels, on day 19, with the same medium and trophic factors as shown in Fig. 1A (supplementary material Fig. S2). After 3 weeks of differentiation, a similar pattern of neocortical neurogenesis as found in the 2D culture conditions was observed. Deep-layer CTIP2^+^ neurons migrated downward from the colonies towards the bottom of the collagen-filled space, with only a few CTIP2^+^ neurons present on the surface of the colonies (supplementary material Fig. S2). Conversely, the majority of upper-layer SATB2^+^ neurons were seen inside the colony, with only a few migrating outwards into the collagen (supplementary material Fig. S2). This 3D cortical model offers an additional way to observe neocortical neurogenesis in a spatially representative manner.

### Premature neuronal differentiation and impaired neocortical patterning induced by cocaine are inhibited by the cytochrome P450 inhibitor cimetidine

**Fig. 4.**
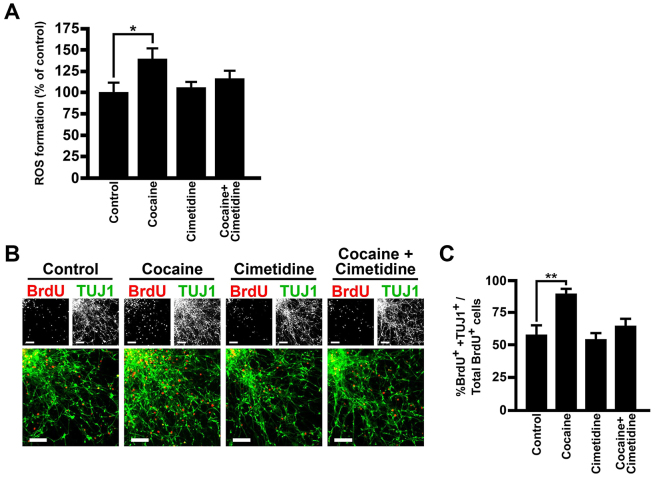
**Cocaine induced ROS generation in neocortical progenitors and premature neuronal differentiation.** (A) ROS formation in neocortical progenitors, at day 19, treated with 3 μM cocaine, for 30 minutes, determined by DCFH-DA; *n*=7. (B,C) Expression of BrdU and TUJ1 (B) by immunocytochemistry in the presence or absence of cocaine and/or cimetidine, at day 27. Percentages of BrdU^+^ and TUJ1^+^ cells of total BrdU^+^ cells are shown in C. Scale bars: 100 μm; *n*=5. *P<0.05, **P<0.01. Error bars, s.e.m. Cell line: H9.

To study how cocaine affects neocorticogenesis and the mechanisms involved in cytochrome-P450-mediated N-oxidative metabolism of cocaine in humans, we first examined whether cocaine induces ROS formation in NE cells derived from hPSCs on day 19. As shown in Fig. 4A, a pharmacologically relevant dose of cocaine, 3 μM (Kourrich et al., 2013), caused a significant increase in ROS 30 minutes after cocaine treatment. Pretreatment of NE cells, 30 minutes prior to cocaine exposure, with the cytochrome P450 inhibitor cimetidine, which blocks the N-oxidative metabolism of cocaine, inhibited cocaine-induced ROS formation (Fig. 4A). We previously reported that cocaine concentrations in the fetal rat brain decrease rapidly less than 1 hour after cocaine injection (Lee et al., 2008). Therefore, in order to create a physiologically meaningful drug treatment schedule *in vitro*, hPSC-derived neocortical cells were treated with 3 μM cocaine for 1 hour at days 20, 22 and 24, the beginning of neocortical neurogenesis, during which the NE cells are actively cycling.

In our neocortical model, post-mitotic neurons migrated outward to the periphery of the colonies, and dividing cells, which expressed Ki67, were found solely in the colony center (supplementary material Fig. S3A). Neurogenesis was examined using BrdU pulse-chase experiments (1-hour BrdU pulse, 24-hour chase) from day 26 to day 27, which showed that the percentage of BrdU^+^ cells that expressed TUJ1 was increased by cocaine as compared with controls (Fig. 4B,C). These data show that cocaine caused premature neuronal differentiation. Although TUJ1 is not solely expressed in post-mitotic cells, only 1.3±0.3% (*n*=3 counted from 6613 cells) of TUJ1^+^ cells in our model expressed Ki67, indicating that the vast majority of TUJ1^+^ neurons in our cultures were no longer dividing. Therefore, proliferating TUJ1 cells should not impact our results. A loss of PAX6^+^ BrdU-positive cells, in conjunction with the increase in TUJ1^+^ BrdU-positive cells, was also observed in cocaine-treated cultures (supplementary material Fig. S3B,C). Pretreatment with cimetidine prevented these effects of cocaine (Fig. 4B,C and supplementary material Fig. S3B,C).

**Fig. 5.**
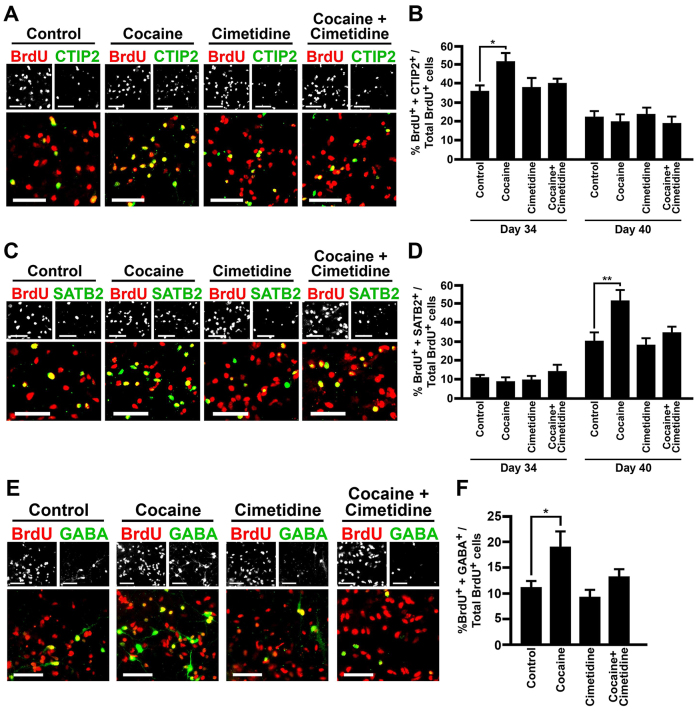
**Cocaine enhanced neurogenesis, for both glutamatergic projection neurons and GABAergic interneurons.** (A) Expression of BrdU and CTIP2 by immunocytochemistry in the presence or absence of cocaine and/or cimetidine, at day 34. (B) Percentages of BrdU^+^ and CTIP2^+^ cells of total BrdU^+^ cells. (C) Expression of BrdU and SATB2 by immunocytochemistry in the presence or absence of cocaine and/or cimetidine, at day 40. (D) Percentages of BrdU^+^ and SATB2^+^ cells of total BrdU^+^ cells. (E) Expression of BrdU and GABA by immunocytochemistry in the presence or absence of cocaine and/or cimetidine, at day 40. (F) Percentages of BrdU^+^ and GABA^+^ cells of total BrdU^+^ cells. Scale bars: 50 μm; *n*=5. *P<0.05, **P<0.01. Error bars, s.e.m. Cell line: H9.

We performed additional BrdU pulse-chase experiments combined with deep-layer and upper-layer cortical markers to analyze whether cocaine-induced premature neuronal differentiation interrupts the subsequent production of cortical neuronal subtypes from day 33 to day 40. In controls, CTIP2^+^ neuron generation peaked at day 34, followed by SATB2^+^ neurons at day 40 (Fig. 5A–D), exhibiting a biologically accurate temporal order. Cocaine, however, significantly increased the production of CTIP2^+^ and SATB2^+^ neurons at day 34 and 40, respectively, during the most active period of neurogenesis for each subtype (Fig. 5A–D). In addition, cocaine significantly increased GABAergic neurons at the end of cell culture (Fig. 5E,F). Each of these cocaine-induced neocortical patterning abnormalities was inhibited by pretreatment with cimetidine (Fig. 5A–F). Taken together, these data suggest that cytochrome-P450-mediated N-oxidative metabolism of cocaine in NE cells induces premature neuronal differentiation and augments cortical neurogenesis.

## DISCUSSION

We have established an hPSC differentiation protocol that uses defined forebrain trophic factors to generate neocortical neurons in a manner that parallels *in utero* forebrain development. The differentiation process retains essential features of human neocortical development, including conversion of hPSCs to a complex population of neocortical progenitors, temporal differentiation of glutamatergic projection neurons, generation of GABAergic interneurons from dorsal forebrain primed NE cells, and development of a radial glial scaffold-based neocortical structure. Although replicating many essential features of neocortical development, our model does not replicate all of the various human neocortical progenitor types, such as outer radial glia (oRG) described by Hansen et al. (Hansen et al., 2010), glial specification steps or specific glial morphologies. A similar neocortical differentiation pattern was observed in the four different human embryonic stem cell (hESC) lines used: H1, H9, ES02 and ES04. This differentiation protocol can be applied in two dimensions, through the use of poly-ornithine/laminin coated plates, or in three dimensions, by embedding the rosettes in collagen gels. The 2D model is advantageous for quantitative experiments, in that it is amenable to measurement and determination of cell numbers.

Previous methods for differentiation of neocortical neurons from hPSCs (Shi et al., 2012; Chambers et al., 2009) did not demonstrate certain features of *in vivo* neocorticogenesis, such as generation of organized radial scaffolding to guide the migration of newly generated neurons. This could be due to the initial dissociation of hPSCs, disrupting intercellular communications such as gap junctions, which have been found in PSCs and play a crucial role in cell differentiation (Oyamada et al., 2013). Additionally, we included bFGF during the development of the neural epithelium, which promotes radial glial scaffolding for later neuronal differentiation. When cultured without bFGF, very little radial glial scaffolding could be observed (data not shown).

In addition, the aforementioned protocols did not generate dorsal-telencephalon-derived GABAergic neuron progenitors, found in the human neocortex *in vivo*. This could be due to the addition of retinoids, in the form of vitamin A, to the cultures, which was used to increase the generation of PAX6^+^ cortical NE cells (Shi et al., 2012). However, retinoic acid (RA), a metabolite of vitamin A, has been detected in the lateral ganglionic eminence (LGE), not the neocortex (Toresson et al., 1999), and is essential in the basal ganglia for the differentiation of LGE-derived GABAergic neurons (Chatzi et al., 2011). Additionally, RA has been shown to induce ventral GABAergic neuronal differentiation from hESCs (Chatzi et al., 2011). Therefore, including RA could guide the differentiation of hPSCs toward a ventral forebrain fate and not mimic *in vivo* human neocortical development.

Shi et al. used retinoids to enhance neocortical neuronal fate, but did not observe GABAergic neurons (Shi et al., 2012). This could be due to differences in protocols: Shi et al. dissociated hPSCs, whereas Chatzi et al. kept hESCs aggregated. Shi et al. also manipulated additional signaling pathways, adding SMAD signaling inhibitors, to achieve rapid neuronal differentiation from ES cells. Our neocortical differentiation system preserves hPSC aggregates, maintaining intercellular communication and the multi-lineage differentiation ability of hPSCs, while employing trophic factors specifically involved in neocortical development. Extrinsic signals involved in differentiation and patterning of other brain regions were excluded to achieve dorsal cortical neuronal differentiation and patterning with a radial scaffold structure.

Even though blocking sonic hedgehog (SHH) signaling is required to generate dorsal telencephalic neurons from mouse ESCs (Gaspard et al., 2008), similar to Li et al. (Li et al., 2009), we found that hPSCs differentiate to a dorsal forebrain identity without inhibition of the SHH pathway. A recent study suggested that inhibition of SHH signaling is crucial for more efficient conversion of hPSCs to dorsal telencephalic progenitor cells (Vazin et al., 2014); however, in the present model we did not see a significant difference in PAX6 expression at the NE stage (day 16) with the addition of the SHH inhibitor, cyclopamine, from day 0 to day 16 (supplementary material Fig. S4). These data suggest that hPSCs have a natural tendency to differentiate to a dorsal phenotype without manipulation of SHH signaling.

It has been shown that there are major species differences in the oxidative metabolism of drugs owing to differences in cytochrome P450 isoforms, limiting extrapolation of data between species (Martignoni et al., 2006). Therefore, an hPSC-based neocortical model might enable more accurate investigations of cortical development as related to human drug and toxin metabolism. We previously showed that, in rats, cytochrome-P450-dependent ROS formation is responsible for cocaine-induced dysregulation of the neural progenitor cell cycle (Lee et al., 2008). Here, we confirmed that cocaine exposure leads to the accumulation of ROS in neocortical progenitors derived from hPSCs and that this increase could be blocked by the cytochrome P450 inhibitor cimetidine. We also provide evidence that cytochrome-P450-dependent ROS generation leads to premature neuronal differentiation, enhanced generation of deep-layer and upper-layer glutamatergic projection neurons, and increased GABAergic interneurons. Cocaine-induced premature neuronal differentiation can help to explain a finding (Lidow et al., 2001) in which [3H]thymidine injections were used to label developing neurons in fetal E90 rhesus monkeys. In controls, labeled neurons appeared in layer III of the cortex, whereas, when mothers were treated with cocaine during the second trimester, [3H]thymidine-labeled cells were randomly distributed in layers III, IV, V, VI, and even in white matter. Additionally, our data substantiates our previous findings showing that cocaine exposure alters glutamate and GABAergic neuron distribution in the developing rat cerebral cortex (Lee et al., 2011).

Overall, our findings suggest that our hPSC *in vitro* culture system can be used to provide more information about cocaine-induced disturbances of neocortical laminar organization. In addition, this is the first time the impact of cocaine on the neurogenesis of various cortical layer neurons, and dorsopallial-derived GABAergic neurons, has been reported. Our model also points to premature neuronal differentiation of cortical progenitors as the reason for these cocaine-induced abnormalities. Our model, including the 3D approach, can be used to study additional neurodevelopmental disorders and provide a more thorough understanding of the pathologies of these disorders.

## MATERIALS AND METHODS

### hESC culture

hESC lines H1 (P42–47) and H9 (P46–53), provided by WiCell Research Institute, and ES02 (P47–56) and ES04 (P67–71), provided by ES Cell International (Singapore), were propagated in feeder-dependent culture, using irradiated mouse embryonic fibroblasts (MEFs; Global Stem). hESCs were cultured in hESC medium, containing DMEM/F12 with 20% Knockout Serum Replacement (KSR), 2 mM L-glutamine, Pen*/*Strep (50 U/ml and μg/ml, respectively), 2 mM nonessential amino acids, 0.1 mM β-mercaptoethanol and 4 ng/ml bFGF (all from Invitrogen). Colonies were passaged using 1 mg/ml Collagenase Type IV (Invitrogen) every 5 days (1:3 split ratio).

### Neocortical neuronal differentiation

hESCs were differentiated to dorsal forebrain cortical neurons as shown schematically in Fig. 1A. Undifferentiated hESC colonies were picked up from feeder-dependent culture using 1 mg/ml collagenase type IV for 30 minutes. hESC aggregates were preserved, allowing EB formation (day 0). EBs were grown, floating, in hESC medium without bFGF for 4 days. The EBs were then transferred to neural media containing DMEM/F-12 (2:1) with N2 supplement, 0.1 mM non-essential amino acids, and 2 μg/ml heparin, supplemented with 20 ng/ml bFGF from days 4 to 6. Colonies were then grown in adherent culture, on laminin-coated plates, in the same media from days 6 to 16. On day 16, dorsal cortical rosettes were isolated by manual dissection and dissociated into 50- to 100-μm-diameter colonies, and maintained in suspended culture with 20 ng/ml bFGF before being seeded on poly-ornithine/laminin-coated dishes at day 19. Once seeded, the colonies were cultured in neuronal differentiation media, containing neurobasal medium, B27 supplement, 0.1 mM non-essential amino acids, 0.5 mM L-glutamine, and 2 μg/ml heparin, supplemented with 20 ng/ml BDNF, FGF18 and NT3 for 3 weeks to generate dorsal forebrain cortical neurons.

### 3D neocortical cultures

At day 19 of differentiation, NE colonies were embedded into 0.5 mg/ml collagen gel in a 96-well plate. 3 mg/ml collagen stock was prepared by dissolving collagen (rat tail collagen, Roche Applied Sciences) in 0.2% acetic acid. This collagen stock (3 mg/ml) was added in a 1:1 volume ratio to 2× PBS, followed by the addition of neuronal differentiation media to make the final 0.5 mg/ml collagen. The pH was neutralized using 1 N NaOH. This collagen solution was then distributed in 150 μl aliquots into wells of a 96-well plate. One NE colony was then added to each well, and incubated for 90 minutes at 37°C to allow the gel to set. 150 μl of neuronal differentiation media was then added on top of the gel, and 50% of media volume was exchanged every other day for 3 weeks.

### Immunocytochemistry and cell counting

Cells were fixed with 4% PFA for 10 minutes, washed with PBS, and blocked with 0.2% Triton X-100 in PBS supplemented with 5% BSA and 10% goat serum. Cells were then incubated with primary antibodies in 0.2% Triton X-100 in PBS with 5% BSA and 5% goat serum: mouse anti-nestin (1:50; R&D Systems), mouse anti-Ki67 (1:100; BD Biosciences), rabbit anti-BF1 (1:100; Abcam), rat anti-HOXB4 (1:50; DSHB), rabbit anti-OTX2 (1:1000; Millipore), mouse anti-NKX2.1 (1:200; Chemicon), rabbit anti-PAX6 (1:300; Covance), mouse anti-Mash1 (1:500; BD Pharmingen), rabbit anti-DLX2 (1:200; Millipore), rabbit anti-NeuN (1:500; Millipore), mouse anti-3CB2 (1:10; DSHB), rabbit anti-glutamate (1:2000; Sigma), rabbit anti-TUJ1 (1:2000; Covance), mouse anti-TUJ1 (1:2000; Promega), rabbit anti-VGluT1 (1:500; Synaptic Systems), rabbit anti-VGluT2 (1:500; Synaptic Systems), rabbit anti-GABA (1:1000; Sigma), mouse anti-GABA (1:100; Sigma), rabbit anti-VGAT (1:500; Synaptic Systems), rat anti-CTIP2 (1:1000; Abcam) and mouse anti-SATB2 (1:200; Santa Cruz Biotechnology). Corresponding fluorescent-labeled secondary antibodies were used (Alexa Fluor 488 for green; Alexa Fluor 555 for red; R&D Systems). Images were captured using a Carl Zeiss Axiovert 200M (Jena, Germany) microscope, and cells in four randomly selected fields each containing 150–750 cells were counted for three to five independent cultures. Data for Fig. 4C (percentage of BrdU^+^ cells that expressed TUJ1), supplementary material Fig. S3C (percentage of BrdU^+^ cells that expressed PAX6) and percentage of TUJ1^+^ cells that expressed Ki67 cell cultures were collected and dissociated into single cells using acutase, prior to being reseeded on poly-ornithine/lamanin-coated plates for quantification. For Fig. 5, cells were quantified in the outside areas of the colonies.

### 3D neocortical culture immunostaining

The neocortical cells wrapped in the collagen gel were immersed in 4% PFA for 20 minutes and cryoprotected in 20% sucrose in PBS for 20 minutes. Cryostat sections (10 μm) were thaw-mounted onto gelatin-subbed slides. Immunocytochemistry protocol was then used, as described above.

### BrdU labeling

Following BrdU (BD Biosciences) incorporation, cells were fixed with 4% PFA for 10 minutes, treated with 95% methanol for 10 minutes, permeabilized with 2 N HCl for 10 minutes, and neutralized twice with 0.1 M sodium borate for 5 minutes each. Cells were then blocked using the immunocytochemistry protocol as described above, with rabbit anti-BrdU (1:200; Rockland) or rabbit anti-BrdU (1:100; Abcam) as the primary antibody.

### Electrophysiology

Cells were recorded at 21°C in a bathing medium composed of (mM): NaCl, 150; KCl, 4; CaCl_2_, 2; MgCl_2_, 1; NaH_2_PO_4_, 0.5; HEPES (hemi-sodium), 10; glucose, 10 (pH 7.4, osmolarity 320 mOsm). The pipette solution for the patch-clamp pipettes consisted of (mM): KCl, 140; MgCl_2_, 2; CaCl_2_, 1; EGTA, 11; HEPES-KOH, 10; ATP (magnesium salt), 4; GTP (sodium salt), 0.4 (pH 7.2). Signals, filtered at 5 kHz and sampled at 25 kHz, were recorded by a VE-2 amplifier (Alembic Instruments, Montreal, Canada) using 100% series resistance compensation, under command of pClamp 7 (Molecular Devices, Sunnyvale, CA).

### Drugs

Cocaine hydrochloride was provided by the National Institute on Drug Abuse. Cimetidine and cyclopamine were obtained from Sigma-Aldrich and Calbiochem, respectively.

### Analysis of endogenous ROS formation

Endogenous ROS levels were measured by incubating NE colonies at day 19 with 100 μM 2′,7′-dichlorofluorescein diacetate (DCFH-DA) (Sigma-Aldrich) for 30 minutes with or without cocaine. The cells were washed and dissolved in 1% Triton X-100 in PBS. Fluorescence for each sample was measured at an excitation wavelength of 485 nm and an emission wavelength of 530 nm using a fluorescence microplate reader (Techan Genios). Protein concentration for each sample was determined using the BCA assay (Pierce BCA^®^ Protein Assay Kits; Thermo Scientific), according to the manufacturer’s instructions. The level of endogenous ROS for each sample was determined by dividing the fluorescence units by the concentration of protein in the lysate.

### RT-qPCR

RT-qPCR was employed to quantify *PAX6* expression using cDNA synthesized from DNase-treated RNA (Transcriptor First Strand cDNA Synthesis Kit; Roche). qPCR was performed and analyzed with the LightCycler 480 Real-Time PCR System (Roche) using LightCycler 480 Probes Master as described previously (Tsai et al., 2012). The primer sequences and probe for PAX6 were 5′-GGCACACACACATTAACACACTT-3′ (Forward) and 5′-GGTGTGTGAGAGCAATTCTCAG-3′ (Reverse), probe #9. These were designed using Universal ProbeLibrary Assay Design Center (Roche) and their specificities were confirmed by standard and melting curve validation. Measurements were performed in duplicate in two separate runs of five independent biological samples, and the results were normalized to a reference gene, *GAPD*. Quantification was performed using the comparative C_T_ method.

### Statistical analysis

Data are shown as means ± s.e.m. Means were compared by two-tailed *t*-tests, or by one-way ANOVA followed by Tukey’s compromise post-hoc test for multiple comparisons, using GraphPad InStat Version 3 software. The criterion for statistical significance was *P*<0.05.

## Supplementary Material

Supplementary Material
