# Supplementary material for: An in vitro model of human neocortical development using pluripotent stem cells: cocaine-induced cytoarchitectural alterations
Source: Dis Model Mech. 2014 Oct 2;7(12):1397–405. doi: 10.1242/dmm.017251 (PMC4257008; doi:10.1242/dmm.017251)
Supplement: Supplementary Material [file supp_7_12_1397__index.html]

An in vitro model of human neocortical development using pluripotent stem cells: cocaine-induced cytoarchitectural alterations — Supplementary Material 

# An *in vitro* model of human neocortical development using pluripotent stem cells: cocaine-induced cytoarchitectural alterations

## DMM017251 Supplementary Material

**Files in this Data Supplement:**

- **Supplementary Material**
