# Supplementary material for: An in vitro model of human neocortical development using pluripotent stem cells: cocaine-induced cytoarchitectural alterations
Source: Dis Model Mech. 2014 Oct 2;7(12):1397–405. doi: 10.1242/dmm.017251 (PMC4257008; doi:10.1242/dmm.017251)
Supplement: Supplementary Material [file supp_7.12.1397_DMM017251.pdf]

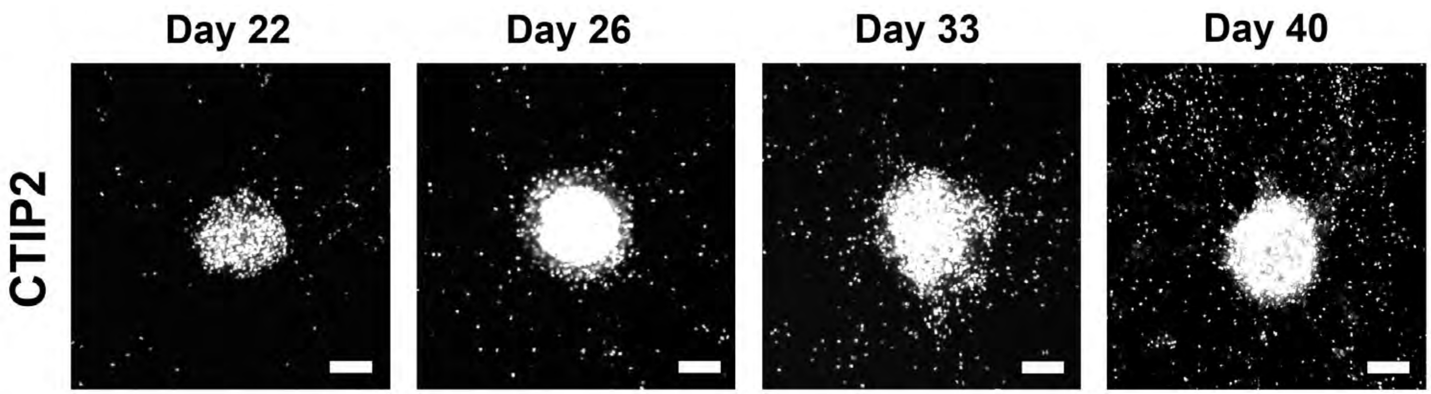

Supplementary Figure 1. Deep-layer CTIP2 neuron generation. Expression of cortical layer marker, CTIP2 by immunocytochemistry from day 22 to day 40 of differentiation. Scale bars: 100 µm. Cell line: H1.

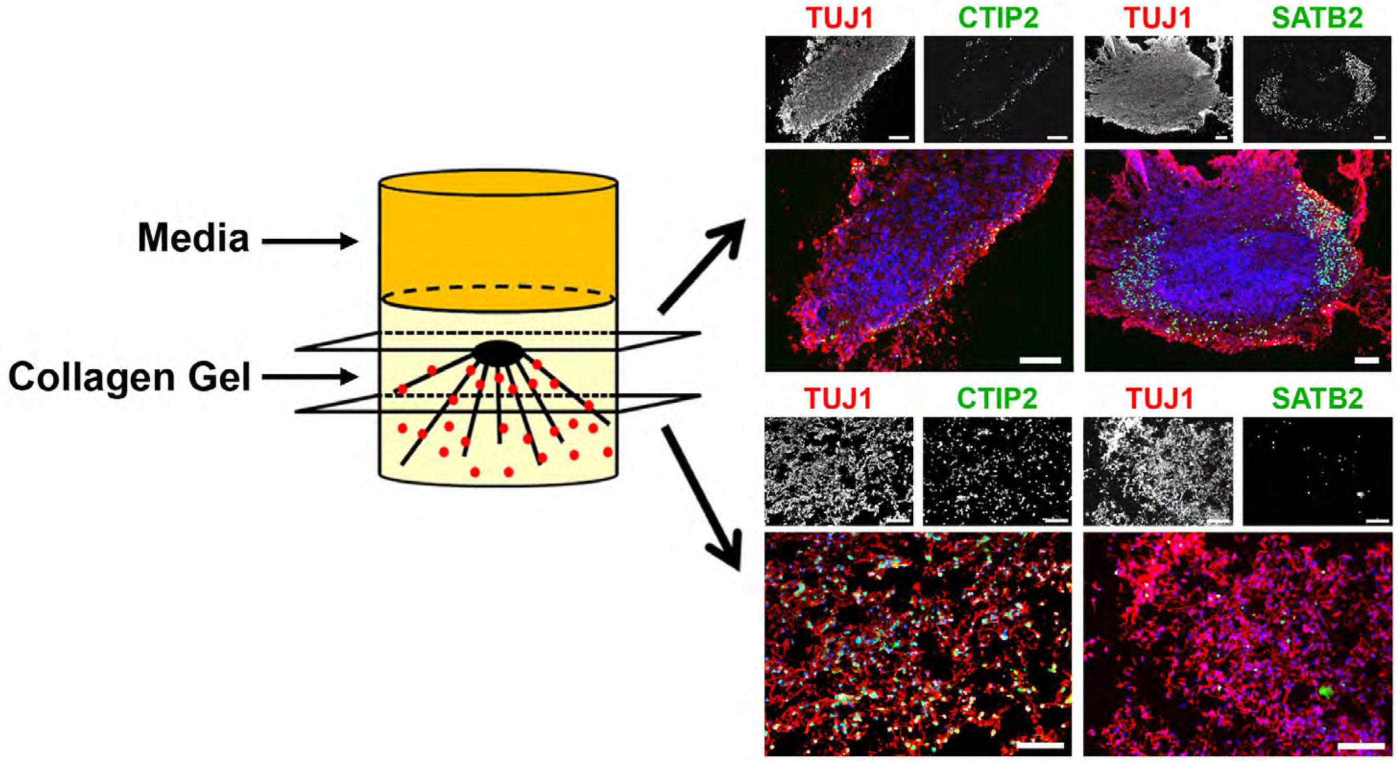

Supplementary Figure 2. 3D neocortical cultures. Dorsal forebrain NE colonies were embedded in collagen gel, at day 19, for 3 additional weeks of differentiation. The upper image represents expression of CTIP2/ TUJ and SATB2 /TUJ1 in colony bodies. The image below displays expression of CTIP2 /TUJ1 and SATB2 /TUJ1 at approximately the middle of the extended scaffolding. Scale bars: 100 µm. Cell line: ES02.

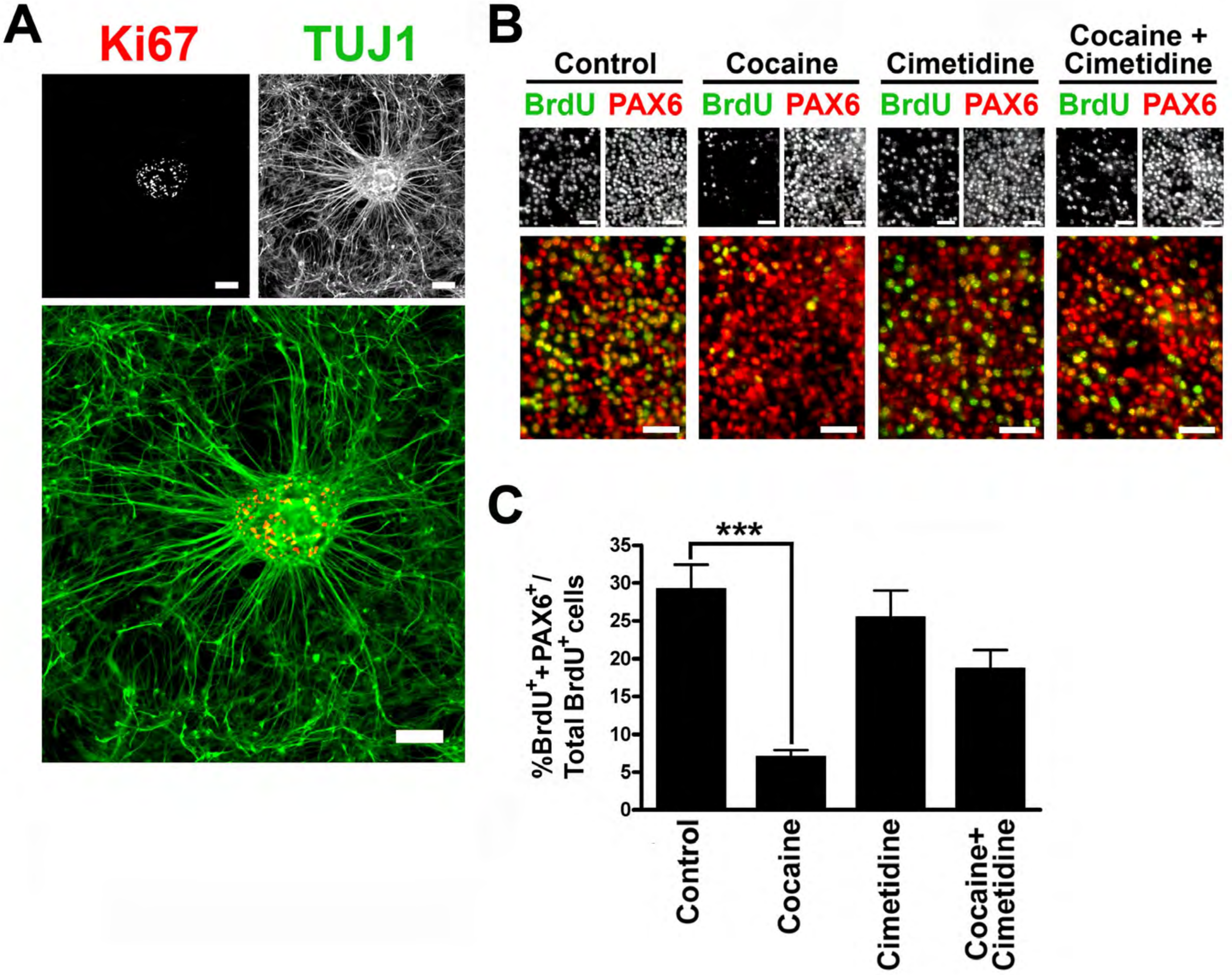

Supplementary Figure 3. Neocortical differentiation and cocaine induced decrease of PAX6$^+$ BrdU-positive cells. A) Expression of Ki67 and TUJ1 by immunocytochemistry at day 26. Scale bars: 100 µm. (B, C) Expression of BrdU and PAX6 (B) by immunocytochemistry in the presence or absence of cocaine and/or cimitedine, at day 27. Percentages of BrdU$^+$ and PAX6$^+$ cells of total BrdU$^+$ cells are shown in (C). Scale bars: 50 µm; n = 5. ***P<0.001. Error bars, s.e.m. Cell line: H9.

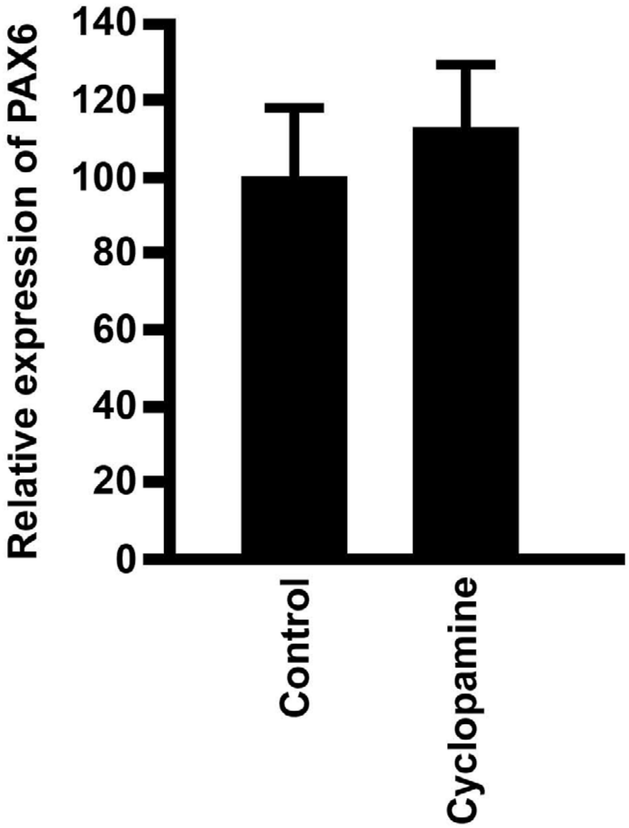

Supplementary Figure 4. Relative expression of *PAX6*. RT-qPCR was used to examine the expression of *PAX6* in neocortical cultures, at day 16, in the presence or absence of 1 μM cyclopamine, a SHH inhibitor, from day 0 to day 16, *n*=5. Cell line: H9.
